# The structure of health in Europe: The relationships between morbidity, functional limitation, and subjective health

**DOI:** 10.1016/j.ssmph.2021.100911

**Published:** 2021-09-06

**Authors:** Aija Duntava, Liubov V. Borisova, Ilkka Henrik Mäkinen

**Affiliations:** Department of Sociology, Uppsala University, Sweden

**Keywords:** Structure of health, Morbidity, Functional limitation, Subjective health, Structural equation modeling, Europe

## Abstract

The main objective of this study is to explore the relationships between the three commonly used proxies of health, morbidity, functional limitation, and subjective health, using the most recent data from 18 European countries. The existing studies on the topic are outdated, limited to the United States and to elderly population. Data on 32,679 respondents of the European Social Survey (2014) were analyzed using structural equation modeling. The results suggest that (a) morbidity and functional limitation lead to poorer self-rated health, and (b) morbidity increases the probability of reporting functional limitation(s). Moreover, functional limitation mediates the relationship between morbidity and self-rated health. The model as a whole holds across both genders and all age groups. However, specific tests (SEM multi-group analyses, *t*-tests) show differences in the health structure between all seven subsamples compared with each other. When both gender and age are taken into account the differences in the structure of health seem to diminish, apart from the elderly, suggesting that the health structure of the elderly differs from others. It is recommended for policy planners to acknowledge the group differences when shaping the policies and health services.

## Highlights

• First study to test a structure of health model on Europeans of all adult ages.
• Morbidity affects functional limitation and subjective health.
• Functional limitation has a negative effect on subjective health.
• Morbidity also affects subjective health indirectly via functional limitation.
• The model is stable across the groups despite some differences in the effect magnitudes.

## 1 Introduction

Health is widely researched across many disciplines and serves as a collection name to represent its different aspects. The most commonly used proxies of health in the field of social epidemiology are mortality, morbidity, functional limitation, and subjective (or ‘self-reported’, “self-rated”) health. There have been many studies investigating the relationships between these concepts. For example, self-rated health has been shown to predict both mortality (DeSalvo et al., 2006; Kaplan & Camacho, 1983; Lee, 2000) and functional limitation (Idler & Kasl, 1995; Lee, 2000), and functional limitation, in turn, has been found to predict self-rated health (Galenkamp et al., 2013; Hays, Schoenfeld, & Blazer, 1996). Likewise, morbidity has been shown to be a significant predictor of subjective health (Galenkamp et al., 2013; Hays et al., 1996). Relationships between specific health conditions and functional decline have also been established in longitudinal studies (Idler & Kasl, 1995; Kriegsman, Deeg, & Stalman, 2004). Moreover, in a longitudinal study Bernard et al. (1997) found that functional limitation along with self-rated health and some health conditions predicts mortality.

However, despite the numerous studies, the full story of these relationships remains to be told. The aforementioned studies sought to establish direct relationships between different aspects of health using only one outcome variable at a time. Seeing that the relationships are more complex, they should, in our opinion, be modeled with both direct and indirect effects between the aspects of health, thus including multiple dependent variables simultaneously. Structural equation modeling (SEM), a multiple-equation technique, is a suitable statistical method to overcome these shortcomings. Using SEM it is possible to investigate how different aspects of health are interrelated to each other in one structure of people's health.

Literature investigating the effects of various social factors on different health outcomes employing SEM is abundant (see, for example, Bardenheier et al. (2013), Santamaría-García et al. (2020), or Wang, Cheng, and Tan (2019), just to mention few). However, the studies exploring the relationships between different aspects of health utilizing SEM date back to the 1980s and 1990s (Gibson, 1991; Johnson & Wolinsky, 1993, 1994; Liang, 1986; Liang et al., 1991; Stump et al., 1997; Whitelaw & Liang, 1991), even though many questions remain unanswered. First, the studies have primarily focused on the *elderly population*, and it is unknown whether the observed relationships hold across all ages. Second, with a few exceptions such as Liang et al. (1991) and Leinonen et al. (1999) (see Note 1 in Supplement), the studies have been conducted in the United States, and it is unclear whether they can be applied elsewhere. In order to extend the scope beyond the United States and to include non-elderly populations, this study investigates the entire adult population in 18 European countries, addressing the following research questions:

(1) What are the relationships between morbidity, functional limitation, and subjective health in the European population?
(2) Does the structure of the observed relationships vary between males and females, and between different age groups in Europe?
(3) Is the strength of the relationships (in terms of their effect size) similar across these groups?

## 2 Background

### 2.1 Previous models of health structure

Various models of health, albeit with different names such as “model of physical health” (Liang, 1986), “model of self-reported physical health” (Whitelaw & Liang, 1991), and “structure of health status” (Johnson and Wolinsky, 1993, Johnson and Wolinsky, 1994), comprising slightly different aspects of health, have been tested by researchers on population data. They all share the common feature of trying to model the structure of health by dividing it into its different aspects and their interrelations. Therefore, they are here referred to as *models of health structure*.

Liang (1986) was the first to propose a health structure model for the elderly population, composed of five latent aspects: existence of chronic illness, number of sick days, physical self-maintenance, mastering instrumental activities of daily living, and subjective health status. “Physical self-maintenance” captured functional abilities such as bathing, walking up and down stairs, and dressing oneself, among others. These abilities were used as predictors of mastering the instrumental activities that reflected a social definition of health, operationalized by such indicators as driving, gardening, etc. Taken together, physical self-maintenance and mastering of instrumental activities reflected different “dimensions” of role performance, the former at basic and the latter at more advanced level. The results indicated that physical illness affects the number of days one is being sick, one's role performance, and subjective health. Sick days influence both self-maintenance and subjective health, while self-maintenance has an effect on instrumental activities, which in turn affects subjective health. Thus, the model was supported, with two exceptions: the effect of sick days on instrumental activities and the effect of self-maintenance on subjective health remain inconclusive.

In an attempt to reduce the complexity of Liang's model (1986), Whitelaw and Liang (1991) introduced a more parsimonious, three-dimensional (see Note 2 in Supplement) model of health consisting of chronic illness, functional limitation, and self-rated health. The results were consistent with Liang's (1986) previous study, indicating that chronic illness has both direct and indirect (through functional limitation) effects on poor self-rated health and a direct effect on functional limitation. In addition, functional limitation has a direct effect on poor self-rated health.

Some years later, a model encompassing disease, disability, functional limitation, and perceived health was developed by Johnson and Wolinsky (1993). The model's causal flow proceeds from disease via disability into functional limitation, reaching perceived health. In general, the hypothetical relationships were confirmed. Diseases were in this model treated separately (instead of forming one latent construct), thus allowing for influence over other aspects of health. Johnson and Wolinsky (1993) also pointed out that disability, rather than disease, affects functional limitation, and that several diseases have their own direct effects on perceived health, such as atherosclerosis (hardening of the arteries), hypertension, and coronary heart disease.

### 2.2 Proposed model of health structure

**Fig. 1:**
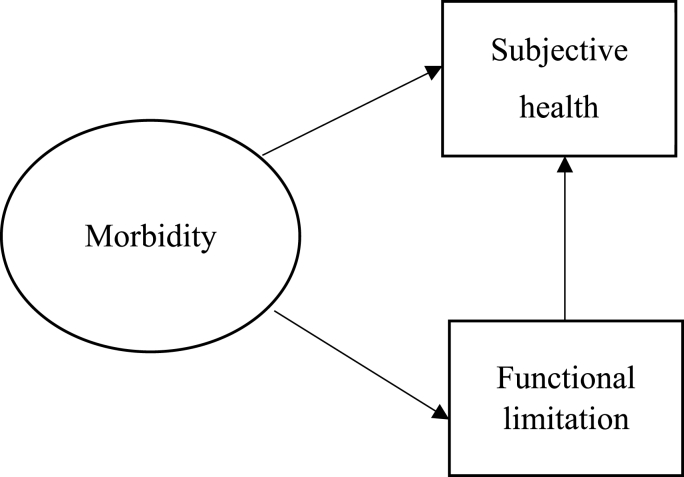
A conceptual model of morbidity, functional limitation, and subjective health. Oval form represents a latent variable, while rectangles represent observed variables. Straight arrows indicate direct effects.

As stated above, health should be studied as an interconnected system, whose parts influence each other and so must be investigated simultaneously. Inspired by the models proposed and tested by Johnson and Wolinsky (1993) and Whitelaw and Liang (1991) described above, an updated model of health structure is proposed here in order to investigate the relationships between different aspects of health. As regards diseases, rather than simply adding various health conditions into indices, alike Whitelaw and Liang (1991), we follow Johnson and Wolinsky (1993) and treat them separately, however only in terms of their direct contribution to the overall morbidity, which appears as a latent variable in this study. Morbidity here is understood as the total effect of different health conditions. This makes it possible to investigate how much of the variation in the single health conditions can be explained by the latent morbidity variable, and also whether the combination of diseases has an effect on functional limitation and self-rated health. Self-rated health and functional limitation are measured by one observed variable each*.* The (simplified) proposed model is illustrated in Fig. 1.

In sum, the model proposed for this study, similar to that by Johnson and Wolinsky (1993), assumes the “natural progression from body to mind” (1993:107), as diseases limit persons in their functional abilities and worsen their perception of well-being. Note that ‘mind’ here does not reflect mental health but rather the subjective *perception* of one's general state of health. In the proposed model, morbidity, placed at the beginning of the causal model, is hypothesized as having a direct effect on both functional limitation and subjective health, and functional limitation is assumed to have a direct effect on subjective health, while serving as a mediator variable for the indirect influence of morbidity on subjective health.

### 2.3 Data and methods

#### 2.3.1 The data set

The data for this research originate from the 7^th^ round of the European Social Survey (ESS) (2014) administrated in 2014. It contained a module called “Social inequalities in health and their determinants”. The current analysis was performed on 18 countries (see Note 3 in Supplement). Only respondents over 18 years of age were included, and cases lacking information on age or gender (see Note 4 in Supplement) were omitted. The remaining sample comprised 33,150 respondents.

### 2.4 Variables

The burden of illness was captured by the construct of *morbidity*. It was measured using the health conditions reported by the respondents answering the question “Which of the health problems […] have you had or experienced in the last 12 months […]?” Respondents were asked about 11 health conditions, here aggregated (after consulting a medical expert) into six binary variables as shown in Table 1.

**Table 1:** The construction of the six indicators of morbidity.

Health conditions in ESS	Indicators created
Heart or circulation problem	Cardiovascular diseases
High blood pressure	
Breathing problems such as asthma attacks, wheezing or whistling breathing	Allergies, respiratory and skin problems (ARS)
Allergies	
Problems related to skin condition	
Muscular or joint pain in hand or arm	Musculoskeletal diseases
Muscular or joint pain in foot or leg	
Severe headaches	Chronic pain
Back or neck pain	
Diabetes	Diabetes
Problems related to stomach or digestion	Gastrointestinal diseases

The question on *functional limitation,* “Are you hampered in your daily activities in any way by any longstanding illness, or disability, infirmity or mental health problem?” had three response alternatives: “yes a lot”, “yes to some extent”, and “no”. These were dichotomized with respondents reporting being very hampered (“yes a lot”) coded as 1, otherwise as 0.

Finally, *subjective health* was operationalized by the question “How is your health in general? Would you say it is…“, with the alternatives “very good”, “good”, “fair”, “bad”, and “very bad”. In order to facilitate comparisons between the three aspects, a dichotomous variable was created, with “bad” and “very bad” coded as 1, others as 0. Thus, “subjective health” rather means “*poor* subjective health”; an increase indicates worsening health.

As one of the objectives of this study is to investigate whether the relationships between the different aspects of health are similar across ages, three age groups (18–44, 45–64, and 65 years or over) were created.

### 2.5 Methods

In evaluating the proposed model, structural equation modeling (SEM) was employed. SEM is a multiple-equation-system technique that offers several advantages (Lei & Wu, 2007). It allows several dependent variables in the analysis. The variables can be latent, and relationships between latent constructs can be estimated. Besides direct effects, indirect effects can be obtained. Thus, entire systems of relationships, including variables that cannot be directly observed, can be analyzed.

SEM consists of two parts, a *measurement* part that evaluates how well the indicators (observed variables) measure the latent construct(s) in the model, while the *structural* part specifies the hypothesized relationships between the variables (Lei & Wu, 2007).

In line with Jöreskog, Olsson and Wallentin (2016), polychoric correlations and their asymptotic covariance matrix with robust diagonally weighted least squares (DWLS) were used. This method is well-suited for analyses with categorical data that often violate the assumptions of normality (Edwards et al., 2012). When employing this method, it is appropriate to report the Satorra-Bentler scaled chi-square (SBχ^2^) value (Yang-Wallentin, Jöreskog, & Luo, 2010) when evaluating the model fit. Additionally, RMSEA (Root Mean Square Error of Approximation), CFI (Comparative Fit Index) and SRMR (Standardized Root Mean Residual) are reported here (see Note 5 in Supplement). Given the very large sample size, the model is considered to be acceptable if it is in the cut-off value range on at least three out of these four goodness-of-fit measures.

#### 2.5.1 Differences in estimates: a multi-group analysis in SEM

Two different methods were employed to investigate group differences. The differences in the parameter estimates across the groups by age and/or gender were tested for both metric and structural invariance in the measurement and structural parts of the model, respectively. *A multi-group comparison* in SEM is the tool for this assessment. In order to test for the invariance (equality) across the groups, a chi-square difference test or likelihood-ratio (LR) test using Satorra-Bentler scaled chi-square (SBχ^2^) values were performed, whereby statistically significant (p<0.05) changes in the SBχ^2^ value mean that the baseline model (with no equality constraint) fits data significantly better than the comparison (with an imposed equality constraint) model. In addition, *t*-tests were performed on the SEM estimates to further assess the between-group differences.

The SEM analysis were performed in LISREL Student Version 9.3 (Jöreskog & Sörbom, 2017), while the data management procedures mentioned above and the *t*-tests were performed in Stata 14.1 (StataCorp, 2015).

## 3 Results

### 3.1 Descriptive statistics

Descriptive statistics and model parameter estimates are reported for 12 groups: (a) the entire sample, (b) males and females, (c) the age groups mentioned above, and (d) six gender-age groups. Descriptive statistics for the health conditions (indicators of morbidity), functional limitation, and self-rated health are presented in Table 2.

**Table 2:** Descriptive statistics on morbidity, functional limitation and subjective health.

Variables	Count	All ages	Males	Females	18–44	45–64	65+	18–44 yrs		45–64 yrs		65+ yrs	
*Morbidity indicators*								Males	Females	Males	Females	Males	Females
Cardiovascular diseases	Yes	8046	3751	4295	885	3080	4081	388	497	1506	1574	1857	2224
	%	24.3	23.9	24.6	6.8	25.9	49.8	6.2	7.3	26.5	25.4	48.9	50.6
Musculoskeletal diseases	Yes	11202	4996	6206	3032	4370	3800	1552	1480	1904	2466	1540	2260
	%	33.8	31.8	35.6	23.2	36.8	46.4	24.9	21.6	33.5	39.8	40.6	51.4
Chronic pain	Yes	14385	6055	8330	5635	5445	3305	2354	3281	2340	3105	1361	1944
	%	43.4	38.6	47.7	43.1	45.8	40.4	37.8	47.9	41.2	50.1	35.9	44.2
Gastrointestinal diseases	Yes	5174	2027	3147	2068	1805	1301	806	1262	692	1113	529	772
	%	15.6	12.9	18	15.8	15.2	15.9	13	18.4	12.2	18	13.9	17.6
Allergies, respiratory and skin diseases	Yes	7564	3292	4272	3105	2553	1906	1385	1720	1050	1503	857	1049
	%	22.8	21	24.5	23.8	21.5	23.3	22.3	25.1	18.5	24.2	22.6	23.9
Diabetes	Yes	1800	906	894	135	637	1028	65	70	352	285	489	539
	%	5.4	5.8	5.1	1	5.4	12.6	1	1	6.2	4.6	12.9	12.3
Functional limitation	Very hampered	2183	931	1252	314	806	1063	133	181	359	447	439	624
	%	6.6	5.9	7.2	2.4	6.8	13	2.1	2.6	6.3	7.2	11.6	14.2
Self-rated health	Poor	2439	980	1459	321	915	1203	114	207	402	513	464	739
	%	7.4	6.2	8.4	2.5	7.7	14.7	1.8	3	7.1	8.3	12.2	16.8

The most common health conditions are chronic pain and musculoskeletal diseases, with 43.4% and 33.8% prevalence. Chronic pain is the most prevalent condition among the younger and middle-aged adults, followed by allergies, respiratory and skin problems (ARS), and musculoskeletal diseases. As expected, cardiovascular problems are the major concern for the elderly population, while musculoskeletal diseases have the second largest prevalence.

The relative prevalence of health conditions is quite similar among both genders of middle-aged adults. Among the younger, chronic pain is the most prevalent health problem for both males and females, with 37.8% and 47.9%, respectively, reporting them. However, musculoskeletal problems are the second largest group of conditions among females, and problems related to ARS among males. As to the elderly respondents, the most dominant health problems are musculoskeletal diseases and cardiovascular diseases among females and cardiovascular diseases among males. Half of the elder males and females (48.9% vs. 50.6%) report cardiovascular problems. On average, females have a higher prevalence of different conditions than males in all age categories, and especially among the elderly.

When it comes to functional limitation, 6.6% of all respondents report being very hampered in their daily activities. This is more often the case with females, and the proportion increases with age. Middle-aged respondents, both males and females, report severe impairments almost three times more often, and elderly respondents more than five times more often than the youngest.

More than 7% of the sample evaluate their health as “bad” or “very bad”. The pattern in relation to gender and age is similar to that for functional limitation.

In sum, the prevalence of most health problems, functional limitation, and negative self-assessment of one's health increases with age, and is greater among females.

The proportion of missing values varies between 0.04% and 1.6% depending on the variable. Listwise deletion was applied, resulting in a sample of 32,679 cases.

### 3.2 Confirmatory factor analysis

First among the analyses was a confirmatory factor analysis (CFA) for the latent variable of morbidity. CFA investigates whether all indicators measure one latent construct. The results of the analysis indicated that the latent construct (morbidity) explained between 16% and 38% of the variance in the indicator variables (see Note 6 in Supplement). All the estimates were statistically significant (p<0.001). However, the initial model fit (SBχ^2^(df)=1611.465(16), p<0.001; RMSEA=0.179; CFI=0.937 and SRMR=0.100) was not good (see Note 5 in Supplement), therefore, the model needed to be re-specified.

Since all six indicators in the CFA model were statistically significant, confirming that they are reliable measures of morbidity, they were retained in the full model. However, the model fit needed improvement. Previous studies have found associations between various diseases, for example, cardiovascular diseases and diabetes (Uusitupa et al., 1985; Wilson, 1998) and between musculoskeletal diseases and chronic pain (Breivik et al., 2006). An association between lower gastrointestinal symptoms and allergic diseases has also been shown (Powell et al., 2007). The association between inflammatory bowel disease (IBD), such as ulcerative colitis (UC) and Crohn's disease (CD) and skin conditions are well described in the review by Keyal, Liu, and Bhatta (2018). Hence, based on the previous research, modification indices, and the model fit evaluation, correlating errors were allowed between (a) cardiovascular diseases and diabetes, (b) musculoskeletal diseases and chronic pain, and (c) gastrointestinal diseases and problems related to allergies, respiration and skin. After this the model fit improved greatly (SBχ^2^(df)=184.307(6), p<0.001; RMSEA=0.084; CFI=0.993 and SRMR=0.0390), being now in the acceptable range.

### 3.3 Structural equation modeling (SEM)

Results from SEM are reported in Table 3 for all 12 groups.

**Table 3:** Parameter and standard error estimates of the model for the whole sample, males, females, and different age groups. All unmarked estimates are significant at the 0.001 level.

	All (T)	Males (M)	Females (F)	18–44 yrs (Y)	45–64 yrs (I)	65+ yrs (E)	18–44 yrs		45–64 yrs		65+ yrs	
							Males (YM)	Females (YF)	Males (IM)	Females (IF)	Males (EM)	Females (EF)
	N=32,679	n=15,491	n=17,188	n=12,935	n=11,706	n=8,038	n=6,162	n=6,773	n=5,603	n=6,103	n=3,726	n=4,312
	Standardized coefficients (standard errors)											
*Measurement part*												
Cardiovascular d.	0.480	0.449	0.512	0.352	0.369	0.416	0.348	0.358	0.347	0.398	0.356	0.463
	(0.010)	(0.016)	(0.014)	(0.024)	(0.018)	(0.019)	(0.039)	(0.031)	(0.027)	(0.024)	(0.029)	(0.026)
Musculoskeletal d.	0.546	0.470	0.602	0.531	0.487	0.490	0.488	0.589	0.449	0.501	0.431	0.524
	(0.010)	(0.016)	(0.014)	(0.024)	(0.018)	(0.020)	(0.038)	(0.031)	(0.027)	(0.024)	(0.030)	(0.027)
Chronic pain	0.391	0.331	0.422	0.647	0.393	0.431	0.597	0.670	0.353	0.409	0.336	0.492
	(0.011)	(0.016)	(0.015)	(0.024)	(0.018)	(0.021)	(0.037)	(0.032)	(0.027)	(0.024)	(0.031)	(0.028)
Gastrointestinal d.	0.392	0.344	0.406	0.570	0.427	0.382	0.535	0.580	0.372	0.452	0.321	0.407
	(0.012)	(0.019)	(0.015)	(0.023)	(0.021)	(0.024)	(0.039)	(0.029)	(0.033)	(0.026)	(0.037)	(0.031)
Allergies, resp., skin diseases	0.350	0.339	0.347	0.406	0.409	0.395	0.394	0.412	0.355	0.436	0.432	0.368
	(0.011)	(0.017)	(0.015)	(0.021)	(0.019)	(0.022)	(0.034)	(0.027)	(0.030)	(0.025)	(0.033)	(0.029)
Diabetes	0.391	0.409	0.392	0.190	0.330	0.302	0.216**	0.182**	0.394	0.292	0.289	0.324
	(0.016)	(0.024)	(0.021)	(0.049)	(0.027)	(0.026)	(0.082)	(0.062)	(0.039)	(0.038)	(0.039)	(0.035)
*Structural part*												
Morbidity - > SH	0.458	0.627	0.375	0.264	0.498	0.317	0.264	0.255	0.612	0.458	0.482	0.241
	(0.035)	(0.079)	(0.039)	(0.042)	(0.061)	(0.053)	(0.069)	(0.054)	(0.118)	(0.077)	(0.124)	(0.058)
Morbidity - > FL	0.766	0.803	0.744	0.518	0.755	0.723	0.492	0.539	0.771	0.757	0.794	0.679
	(0.015)	(0.026)	(0.019)	(0.033)	(0.027)	(0.027)	(0.055)	(0.042)	(0.045)	(0.034)	(0.046)	(0.034)
FL - > SH	0.461	0.308	0.531	0.641	0.435	0.544	0.659	0.634	0.338**	0.465	0.389**	0.609
	(0.033)	(0.077)	(0.038)	(0.036)	(0.058)	(0.049)	(0.055)	(0.049)	(0.114)	(0.073)	(0.118)	(0.056)
*SBχ*^*2*^*(df)*	*343.492(16), p<0.001*	*136.355(16), p<0.001*	*194.497(16), p<0.001*	*60.92(16), p<0.001*	*78.290(16), p<0.001*	*74.292(16), p<0.001*	*50.891(16), p<0.001*	*20.890(16), p=0.183*	*38.398(16), p=0.001*	*48.466(16), p<0.001*	*35.065(16), p=0.004*	*50.532(16), p<0.001*
*RMSEA (CI)*	*0.085*	*0.078*	*0.088*	*0.094*	*0.066*	*0.059*	*0.129*	*0.074*	*0.065*	*0.073*	*0.059*	*0.066*
	*(0.082; 0.087)*	*(0.075; 0.081)*	*(0.084; 0.091)*	*(0.090; 0.097)*	*(0.062; 0.070)*	*(0.055; 0.064)*	*(0.123; 0.134)*	*(0.069; 0.079)*	*(0.060; 0.071)*	*(0.068; 0.079)*	*(0.052; 0.066)*	*(0.060; 0.072)*
*SRMR*	*0.053*	*0.049*	*0.054*	*0.060*	*0.039*	*0.031*	*0.087*	*0.042*	*0.036*	*0.040*	*0.032*	*0.032*
*CFI*	*0.996*	*0.997*	*0.996*	*0.998*	*0.998*	*0.996*	*0.997*	*1.000*	*0.998*	*0.998*	*0.997*	*0.996*

SBχ^2^ - Satorra-Bentler scaled chi-square. RMSEA – Root Mean Square Error of Approximation. SRMR - Standardized Root Mean Residual. CFI – Comparative Fit Index. SH – Subjective health. FL – Functional limitation. Df – degrees of freedom. **- significant at the 0.01 level. Standard errors reported in parentheses. The error variances (not shown) are allowed to correlate between chronic pain and musculoskeletal diseases, between gastrointestinal diseases and problems related to allergies, respiration and skin, and between cardiovascular diseases and diabetes. All the error correlations are statistically significant at least at the 0.05 level, apart from those between gastrointestinal diseases and problems related to allergies, respiration and skin for the youngest males, and those between chronic pain and musculoskeletal diseases and between cardiovascular diseases and diabetes for the youngest females.

Furthermore, the subgroups, based on gender, age, and gender-age, are also compared (see Note 7 in Supplement) regarding differences in both the measurement and structural parts.

#### 3.3.1 The measurement part: relationships between diseases and morbidity

The measurement part of the model quantifies how well the indicators are measuring the latent concept of morbidity. The magnitude of the indicators shows how much of the variation in morbidity can be explained by each type of disease. In the measurement part of the model (Table 3), the results for the total sample (see the model T in Table 3) and for male (model M) and female (model F) subsamples agree as regards the best indicators of morbidity, i.e. musculoskeletal and cardiovascular diseases. However, there are variations in this regard in the other nine subsamples (models Y-EF).

In the young (Y, YM, YF) and middle-aged (I, IM, IF; see Note 8 in Supplement) subsamples, and also among elderly females (EF), the best predictors of morbidity are chronic pain and musculoskeletal diseases. Among the elderly males (EM) the best predictor is the diseases related to allergies, respiration and skin (ARS). The parameter estimates are generally higher for females than for males, implying that the indicators measuring morbidity work better for females. There is no clear pattern of the magnitudes of the predictors in the different age groups.

The group-wise differences in the parameter estimates in the measurement part of the model speak for testing for invariance. Invariance means that there are no statistically significant differences in the parameter estimates between the groups. The metric invariance model is compared with configural invariance model (baseline model, where factor loadings are freely estimated for each group).

*Metric* invariance restricts the factor loadings to be equal across the groups (Millsap & Olivera-Aguilar, 2012). The results for the metric invariance model tests are included in Table 4, and they indicate non-invariance for all seven pairs of groups. Next, each factor loading was tested separately between the subgroups by independent-samples *t*-tests (see Note 9 in Supplement), whereby a number of differences in factor loadings between the groups were found (see Table 5).

**Table 4:** Model comparisons between subgroups.

Groups and models	SB scaled χ^2^	df	Model comparison	Δ SB scaled χ^2^	Δ df	p-value
*Males vs females*						
Configural invariance model (1)	287.638	32	NA			
Metric invariance model (2)	401.983	38	2 vs 1	114.345	6	0.000
Structural invariance (3)	428.432	41	3 vs 2	26.449	3	0.000
*Young adults vs middle-aged adults*						
Configural invariance model (1)	116.045	32	NA			
Metric invariance model (2)	148.159	38	2 vs 1	32.114	6	0.000
Structural invariance (3)	158.465	41	3 vs 2	10.306	3	0.016
*Young adults vs elderly*						
Configural invariance model (1)	98.772	32	NA			
Metric invariance model (2)	121.686	38	2 vs 1	22.914	6	0.001
Structural invariance (3)	139.868	41	3 vs 2	18.182	3	0.000
*Middle-aged adults vs elderly*						
Configural invariance model (1)	132.041	32	NA			
Metric invariance model (2)	158.557	38	2 vs 1	26.516	6	0.000
Structural invariance (3)	205.246	41	3 vs 2	46.689	3	0.000
*Young males vs young females*						
Configural invariance model (1)	106.819	32	NA			
Metric invariance model (2)	130.098	38	2 vs 1	23.279	6	0.001
Structural invariance (3)	145.243	41	3 vs 2	15.145	3	0.002
*Middle-aged males vs middle-aged females*						
Configural invariance model (1)	80.217	32	NA			
Metric invariance model (2)	119.478	38	2 vs 1	39.261	6	0.000
Structural invariance (3)	129.232	41	3 vs 2	9.754	3	0.021
*Elderly males vs elderly females*						
Configural invariance model (1)	75.636	32	NA			
Metric invariance model (2)	113.944	38	2 vs 1	38.308	6	0.000
Structural invariance (3)	122.425	41	3 vs 2	8.481	3	0.037

SBχ^2^ - Satorra-Bentler scaled chi-square. Configural invariance model (1) is one that does not impose any constrains on the parameters. Metric invariance model (2) is a more restrictive model, imposing constrains on factor loadings for morbidity variable to be equal across the groups compared. Factor loadings for functional limitation and subjective health are constrained to be equal to 1. Structural invariance model (3) is the most restrictive model, imposing constraints on both factor loadings for morbidity variable and the path coefficients in the structural part of the model. Factor loadings for functional limitation and subjective health are constrained to be equal to 1.

**Table 5:** A summary table of independent-samples *t*-test results for differences in individual SEM estimates between different groups.

Males vs females	Young vs middle-aged	Young vs elderly	Middle-aged vs elderly	Young males vs young females	Middle-aged males vs middle-aged females	Elderly males vs elderly females
*Measurement coefficients*						
Cardiovascular diseases	Chronic pain	Chronic pain		Musculoskeletal diseases	ARS	Cardiovascular diseases
Musculoskeletal diseases	Gastrointestinal problems	Gastrointestinal problems				Musculoskeletal diseases
Chronic pain	Diabetes					Chronic pain
Gastrointestinal problems						
*Structural coefficients*						
M on SH	M on SH	M on FL	M on SH			M on FL
FL on SH	FL on SH					
	M on FL					

M – Morbidity. SH – Subjective health. FL – Functional limitation. ARS – Allergies, respiratory and skin problems.

Significant differences (p<0.05) were found for the parameters included in the table.

#### 3.3.2 The structural part: relationships between the three aspects of health

**Fig. 2:**
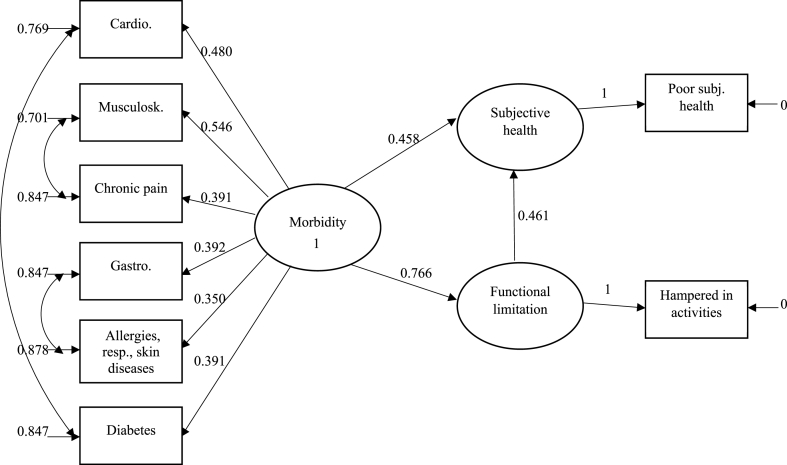
Standardized parameter estimates for the relationships between morbidity, functional limitation, and subjective health among 32,679 European adults. Oval forms represent latent variables, while rectangles represent observed variables. Straight arrows represent direct effects. Double-headed curved arrows represent correlations. All estimates are significant at the 0.001 level. Correlations between error variances (not shown) are all positive and statistically significant at the 0.001 level.

The analysis of the structural part of the model (see Fig. 2) shows that morbidity has a statistically significant, direct effect on both subjective health and functional limitation at the individual level. An increase in morbidity leads to a higher probability of both evaluating one's health negatively (B=0.458, p<0.001) and being very hampered in one's daily activities (B=0.766, p<0.001). Functional limitation in turn influences subjective health directly: being very hampered in one's daily activities leads to a higher probability for evaluating one's health negatively compared to those respondents who are less or not at all hampered, even when the effect of morbidity on subjective health is controlled for (B=0.461, p<0.001).

The estimates differ somewhat between males and females (see the models M and F in Table 3). The effect of morbidity on subjective health is higher for males (B=0.627, p<0.001) than for females (B=0.375, p<0.001). The effect of morbidity on functional limitation is the strongest relationship for both males (B=0.803, p<0.001) and females (B=0.744, p<0.001), and functional limitation affects females' subjective health stronger (B=0.531, p<0.001) than males’ (B=0.308, p<0.001).

An analysis for the three age groups (Y, I and E) shows that the path estimates differ somewhat, but not in a consistent fashion. The effect of morbidity on subjective health is lower for the youngest and the oldest respondents (B=0.264, p<0.001 and B=0.317, p<0.001, respectively), and higher for the middle-aged (B=0.498, p<0.001). The effect of morbidity on functional limitation is highest among the middle-aged (B=0.755, p<0.001) and the elderly (B=0.723, p<0.001), however, functional limitation affects subjective health most strongly in the youngest age group (B=0.641, p<0.001). In general, the estimates are higher for the middle-aged (I) in terms of the effect of morbidity on both subjective health and functional limitation.

The analysis was also performed for the same age groups divided by gender. The estimates for the youngest age group are quite similar for both genders (YM and YF), while there are gender differences in other age groups. Morbidity affects males' subjective health more than females’ in both of the older age groups.

So far, only direct effects have been presented. However, the proposed model also assumes an indirect effect of morbidity on subjective health via functional limitation. When testing this, the results (available on request) for the entire sample suggest that a substantial part of the effect of morbidity on subjective health (B=0.811, p<0.001) is indirect, passing through functional limitation (B=0.353, p<0.001). The proportion of the indirect effect varies from 28.3% to 63.1% between subsamples. The indirect effects are statistically significant (p<0.001) in the total sample and all subsamples.

Having observed some group-wise differences in the structural part, the next step was to test whether the path coefficients between variables differ between groups. The results arising from a structural invariance model were compared with those from the metric invariance model (see Table 4). The LR test indicates non-invariance (ΔSBχ^2^(df) is significant), suggesting differences to exist between all groups compared. The independent-samples *t*-tests on the individual structural coefficients (see Table 5) indicate eight differences in path coefficients between different groups.

#### 3.3.3 Model fit

The model for the entire sample, and those for all age and/or gender subsamples, show an acceptable fit (RMSEA<0.1; CFI>0.9 and SRMR<0.10), with the exception of the model for the youngest males, which failed on SBχ^2^ and RMSEA measures (SBχ^2^(df)=50.891(16), p<0.001; RMSEA=0.129; CFI=0.997 and SRMR=0.087). The model for young females, on the contrary, fit the data by all measures (SBχ^2^(df)=50.891(16), p=0.183; RMSEA=0.074; CFI=1; SRMR=0.042). The models for the elderly respondents (SBχ^2^(df)=74.292(16), p<0.001; RMSEA=0.059; CFI=0.996 and SRMR=0.031) and for elderly males (SBχ^2^(df)=35.065(16), p=0.004; RMSEA=0.059; CFI=0.997 and SRMR=0.032) show a good fit on three measures. Overall, considering the number of models run and the sample and subsample sizes the fit can be considered acceptable.

## 4 Discussion

### 4.1 The model

The results of this study show that having more health problems and being very hampered in one's daily activities both independently increase the probability of individuals in European adult population to evaluate their health negatively. Having more health problems also directly affects functional limitation by increasing the probability of reporting being very hampered in one's daily activities. The observed, statistically significant, indirect effect of morbidity on subjective health via functional limitation implies that health problems also affect the evaluation of one's health through difficulties in performing activities of daily living, with worsening quality of life as a consequence. These results agree with those of Whitelaw and Liang (1991) in the USA, drawing our attention to the fact that both health conditions and the ability to perform daily activities affect one's subjective evaluation of health. In sum, the proposed model and the specified relationships (see Fig. 1) were confirmed*.* The same relationships were also confirmed among the subgroups of males and females, different age groups, and the gender-age groups. Most of the models were deemed as acceptable, with the exception of young males.

The observed relationships suggest that having bodily problems influences one's experience and performance irrespective of age and gender. Of course, physical vulnerabilities may also come with a greater risk of encountering other vulnerabilities of, for example, familial and economic nature.

In general, the proposed model supports the adopted hypothesis of a “natural progression from body to mind” (Johnson & Wolinsky, 1993:107), which suggests that biological conditions in human body are reflected in both physical functioning and the performance of social roles that require such functioning. The presence of these factors manifests itself in the evaluation of subjective health. While the resulting model is certainly not the only one possible – access to more data could have helped in building more complex ones – it fit the existing data and it can provide an economic starting point for improvement.

### 4.2 The construct of morbidity

In this study, morbidity was defined as an accumulation of different diseases, and operationalized by different health conditions being aggregated into groups on a latent variable, which reflects the *total burden of disease* of an individual. Importantly for the model, the indicators of morbidity and the variable functional limitation are not identical in the sense that the captured health problems would necessarily lead to functional limitations in themselves. Thus, the relationship between morbidity and functional limitation is not self-explanatory. A comparison with previous studies concerning the effect of morbidity on other aspects of health yields no conclusive results. While some studies (Liang, 1986; Liang et al., 1991; Whitelaw & Liang, 1991) found relationships between morbidity and other aspects of health, others did so only partly (Johnson & Wolinsky, 1993, 1994). For example, Johnson and Wolinsky (1993), who treated diseases separately, found that only some diseases have direct effects on subjective health, while others mostly affect functional limitation and/or disability. In general, it was shown by them that most diseases influence subjective health only indirectly through disability and functional limitation.

The difference in results reflects the manner in which morbidity was operationalized in the studies. Those studies that found relationships between morbidity and other aspects of health used a latent morbidity variable, while those that did not provide conclusive evidence used variables for various diseases directly.

Is the use of a latent variable a limitation or a strength? One of the aims of this study was to investigate the effect that a disease *burden* might have on other aspects of health, rather than that of any single disease. Measuring individual morbidity, rather than illnesses as such, is a different approach, one that studies “sickliness”, and, indirectly, individual vulnerability in general. Clearly the effects of diseases on functional limitation or subjective health might vary, nevertheless, as shown here, all the disease groups tested contributed to the latent morbidity construct. The results of this study show that this definition of morbidity can be successfully employed.

Apart from physical health conditions, both functional limitation and subjective health can be influenced by mental problems. However, none of the indicators of the latent morbidity variable could capture these, which made the concept of morbidity more narrow here. Future research will hopefully be able to compare different “morbidity” variables – the findings from such studies could be useful for studies of health in general.

### 4.3 Group differences: gender

The multi-group comparison in SEM suggests difference(s) in factor loadings between males and females. The *t*-test results further indicate gender differences in the factor loadings of cardiovascular and musculoskeletal diseases, chronic pain, and gastrointestinal problems. Previously, Johnson and Wolinsky (1994) have shown that gender differences in the measurement part of some health concepts prevail.

An explanation for the gender differences in morbidity indicators found in this study might lie in the subjective evaluation of the severity and duration of the symptom(s), and/or in differences in the process of recognizing health problems in general. The review by Gutiérrez Lombana and Gutiérrez Vidal (2012) summarizes that females report pain more often and have a lower pain threshold compared to males. Moreover, females report more musculoskeletal and neuropathic pain. This would be in line with our findings indicating that musculoskeletal disease is a significantly better indicator of morbidity among females than males in all but one gender-divided subgroups. Moreover, all morbidity indicator loadings with significant gender differences are higher for females, meaning that they have higher ability of capturing the corresponding health problems for females than for males.

As to structural coefficients, the SEM multi-group analysis indicates gender difference(s) in them, too, and the *t*-test results show that they exist in the effects of both morbidity and functional limitation on subjective health. The direct effect of morbidity on subjective health is higher for males while the effect of functional limitation on subjective health is higher for females. The gender differences in reporting health problems could explain the differences in the former effect, where, on average, females’ reporting of more health problems does not lead to poorer evaluation of subjective health compared to males. The explanation for the latter effect might lie in the severity of functional limitation. Merrill et al. (1997) in their study showed that elderly females had poorer performance on all tasks related to disability and functional limitation compared to males. They concluded that, in general, both genders are accurate in reporting their functional problems in relation to the task performance, and that the difference is real. Thus, assuming that females reporting functional limitation suffer more from it than males, it is not surprising to find that functional limitation in females is reflected in poorer evaluation of subjective health.

When exploring the structure of health in the three pairs of gender-age subgroups, the results in the structural part of the model in two of them are inconclusive. The LR tests suggested difference(s) in the coefficient(s) between young males and young females and between middle-aged males and middle-aged females, but the *t*-tests did not support these results. Therefore, further research is needed to investigate whether or not there are differences between these groups.

Taking together the group-wise differences in both measurement and structural parts of the model seem to build a pattern. The number of differences in the health structure between males and females tends to diminish when age is accounted for, with the exception of the elderly. This might indicate that the health structure of the elderly is in this respect different from that of the younger adults. Especially, the effect of morbidity on functional limitation is higher for elderly males than for elderly females. This finding seems interesting considering that females, on average, have higher morbidity scores compared to males, which should lead to a higher probability of having functional limitations. However, this is not the case. This “paradox” reflects perhaps a gender difference in reporting, where females might report health problems that do not translate into higher probabilities of affecting other aspects of health. The explanation to this could lie in how health conditions are being recognized by male and female respondents. What is understood by “daily activities” might as well differ between elderly males and females.

### 4.4 Group differences: age

The LR tests show that age-related differences in factor loadings exist between three pairs of groups. The *t*-tests indicate that the differences lie in the loadings of chronic pain and gastrointestinal problems between the young and the middle-aged and between the young and the elderly, and in those of diabetes between the young and the middle-aged.

One explanation for these differences could be that respondents, due to experience, use different frames of reference when thinking of their health. For example, when a health problem resembling that of the respondent has been experienced by age peers it might be less likely to be acknowledged by the respondent than it would if only few of the age peers would have experienced it. Indeed, Krause and Jay (1994) have shown that when their respondents answered a question about their general health the evoked frame of reference differed by age: young adults (14–24) referred to their own health-related behaviors (exercising, smoking, drinking, etc.) more often than did the elderly (60+).

As regards the path coefficients, the LR test shows differences between all three pairs of age groups. The *t*-tests indicate that all path coefficients differ considerably between the young and the middle-aged, while only the coefficients of the effect of morbidity on functional limitation and of morbidity on subjective health differ between the young and the elderly and between the middle-aged and the elderly, respectively. This might suggest that different mechanisms exist in how the aspects of health are interrelated as individuals are aging.

One explanation can be tied to the psychological acceptance of functional limitations in younger age. Different health conditions might be more easily accepted by the younger adults as they are still young and relatively healthy, thus, existing conditions would not cause much disturbance as they would at a later stage of life. However, being very hampered in one's daily activities can be harder to accept when young, possibly leading to depressive moods and a more negative evaluation of one's general health. Indeed, the effect of functional limitation on subjective health is strongest in the young, while the effect of morbidity on both functional limitation and subjective health is weakest among them. Age appears to make the respondents more vulnerable when it comes to how morbidity influences the other aspects of health, while the opposite is the case for the influence of functional limitation on subjective health. For example, the study by Choi and Kim (2007), although with a far older sample, found that younger elderly persons (55–64) having at least one impairment faced a greater risk of depression compared to older elderly persons (75+) with the same impairment(s).

In sum, considering both parts of the model together, the structure of health of the middle-aged and the elderly appears quite similar, while the younger age group deviates from others.

### 4.5 A universal model?

Overall, it appears that, based on both this study and its predecessors, the relationships between the investigated aspects of health – morbidity, functional limitation, and subjective health – hold for large parts of the developed world such as the United States, Japan, and Europe. If we compare our results with those from other countries (the United States and Japan) where similar models have been tested, we can even speculate whether this type of health model could apply across cultures as well. Yet, the possible diversity between European countries was not controlled for in this study in order not to over-extend the analysis.

The proposed model in this study can be seen as the very first empirical step in the attempt to develop a general health model for the entire adult population, which can serve as a basis for further improvement and group-wise modifications. In this manner this study would constitute a part of the larger work whose results can be practically utilized by both policy planners and practitioners.

## 5 Conclusions

The basic objective of this study was to investigate the relationships between different aspects of health in European countries, including the entire adult population. The results confirm the proposed model and the suggested relationships between the three aspects of health investigated. The model holds for the target population (from 18 European countries), and also for the majority of subsamples within it, despite the differences found between some of the groups.

Significant gender and/or age differences were observed in the magnitudes of effects between all seven pairs of groups. The pattern in these differences was identified when both age and gender were accounted for – the differences in the structure of health between all gender-age groups seem to disappear, apart from elderly males and females. Instead of trying to explain away these differences, future research, health promotion in general, and policies targeting specific population groups in particular would benefit from acknowledging them.

## Funding

This study was funded by the Swedish Research Council's Grant No. 2014–1511 to the project “The different aspects of health: relations between mortality, morbidity, functional status and subjective health in European populations”. The funding source had no involvement and no influence in any stage of conducting this research as well as writing and publishing the article.

## Acknowledgements

We thank the Welfare Research Group at the Department of Sociology at Uppsala University for the valuable comments and support as this article was being developed. This research was made possible thanks to the funding provided by the Swedish Research Council.

## Research data

The data that support the findings of this study are available in ESS7 - integrated file, Edition 2.0 (currently 2.2) at doi:10.21338/NSD-ESS7-2014. These data were derived from the following resources available in the public domain: https://www.europeansocialsurvey.org/download.html?file=ESS7e02_2&y=2014.

## CRediT authorship contribution statement

**Aija Duntava:** Conceptualization, Methodology, Formal analysis, Investigation, Data curation, Writing – original draft, Writing – review & editing, Visualization. **Liubov V. Borisova:** Conceptualization, Methodology, Writing – review & editing, Supervision, Funding acquisition. **Ilkka Henrik Mäkinen:** Conceptualization, Methodology, Writing – review & editing, Supervision, Project administration, Funding acquisition.

## Declaration of competing interest

None.
