# Supplementary material for: The structure of health in Europe: The relationships between morbidity, functional limitation, and subjective health
Source: SSM Popul Health. 2021 Sep 6;16:100911. doi: 10.1016/j.ssmph.2021.100911 (PMC8502770; doi:10.1016/j.ssmph.2021.100911)
Supplement: Multimedia component 1 [file mmc1.docx]

# Supplement: Notes

1. The statistical method employed in their study was path analysis, though.
2. “Health dimensions” in Whitelaw and Liang’s study (1991) reflect the “aspects of health” in this paper.
3. Austria, Belgium, Czech Republic, Denmark, Finland, France, Germany, Hungary, Ireland, Lithuania, Netherlands, Norway, Poland, Slovenia, Spain, Sweden, Switzerland, and United Kingdom.
4. The respondents’ own answer to the question on gender, rather than the interviewer’s report, is used.
5. RMSEA, CFI, and chi-square are the most commonly reported measures when employing SEM. Lai and Green (2016) summarize previous research showing that RMSEA values lower than 0.05 (or 0.06), between 0.05/0.06 and 0.1, and above 0.1 indicate “good”, “acceptable” and “bad” fit, respectively. As to CFI, values above 0.90 indicate an acceptable fit (McDonald and Ho 2002). Even standardized root mean square residuals (SRMR), that measure the average difference between the observed and the model-predicted correlations, where values of less than or equals 0.10 would indicate a good fit (Kline 2016), were taken into account.
6. Due to limitations of space, the results are not presented here but are available on request.

The groups were compared pairwise as follows: (a) males and females, (b) young adults and middle-aged adults, (c) young adults and elderly, (d) middle-aged adults and elderly, (e) young males and young females, (f) middle-aged males and middle-aged females, and (g) elderly males and elderly females.

The subsample of middle-aged adults in this study is abbreviated as “I”, which stands for “intermediate” group.

1. We thank an anonymous reviewer for the suggestion to perform independent-samples *t*-tests.

**References**

Kline, R.B. (2016). Principles and Practice of structural equation modeling. New York: The

Guilford Press.

Lai, K., & Green, S.B. (2016). The problem with having two watches: Assessment of fit when

RMSEA and CFI disagree. Multivariate Behavioral Research, 51(2–3), 220–239.

doi:10.1080/00273171.2015.1134306.

McDonald, R.P, & Ho, M.H.R. (2002). Principles and practice in reporting structural equation

analyses. Psychological Methods, 7(1), 64–

82. <http://dx.doi.org.ezp.sub.su.se/10.1037/1082-989X.7.1.64>.

Whitelaw, N.A., & Liang, J. (1991). The structure of the OARS physical health

measures. Medical Care, 29(4), 332–347.
